# Outcomes of Surgical Treatment of Peripheral Neuromas of the Hand and Forearm

**DOI:** 10.1055/s-0043-1767673

**Published:** 2023-04-05

**Authors:** Yousif Tarek El-Gammal, Laura Cardenas-Mateus, Tsu Min Tsai

**Affiliations:** 1 Christine M. Kleinert Institute for Hand and Microsurgery, Louisville, Kentucky, United States; 2 Department of Orthopedics and Traumatology, Assiut University, Assiut, Egypt; 3 Department of Orthopedics and Traumatology, Hospital Santa Clara, Bogota, Colombia

**Keywords:** neuroma, resection, reconstruction

## Abstract

The choice of a specific technique for surgical treatment of neuromas remains a problem. The purpose of this study is to determine the overall effectiveness of surgery as well as to find out whether certain surgical procedures are more effective than others. Twenty-nine patients operated between 1998 and 2018 and followed for at least 12 months were reviewed. Clinical assessment included the identification of a pre- and postoperative Tinel sign, pain visual analog score, two-point discrimination (2PD), and grip strength. Mechanisms of injury included clean lacerations (11), crush injuries (11), and other trauma or surgery (7). Mean time from presentation to surgery was 9 months. Seven surgical procedures involving excision in 10 patients and excision and nerve repair in 19 patients were performed. Pain score improved from an average of 7.1 ± 2.3 to 1.8 ± 1.7 with 27 patients (93%) reporting mild or no postoperative pain. Nine patients complained of residual scar hypersensitivity and six patients had residual positive Tinel. No patient required an additional surgical procedure. 2PD improved from an average of 9.6 ± 4.0 to 6.8 ± 1.0. The improvement of pain score and 2PD was statistically significant. Nerve repair resulted in marginally better outcomes, in terms of 2PD and grip strength recovery, than excision alone. The mechanism of injury, zone of involvement, time to intervention, or length of follow-up did not have an impact on the outcomes. Although patient numbers in this study are large in comparison to previous studies, larger patient numbers will allow for a multivariate analysis, which can be possible with a prospective multicenter trial.

## Introduction

Neuromas are globular swellings that form as a result of abnormal and disorganized regeneration of unmyelinated nerve endings. 1 2 3 Neuromas can be painful due to either persistent mechanical or chemical irritation of the axons within the neuroma or development of spontaneous activity of neurons within the dorsal root ganglion. 4 Painful neuroma usually develops following nerve trauma or surgery, affecting 2 to 60% of patients with nerve injuries. 5 6 7

Over 150 treatment methods have been reported to control pain caused by neuroma formation; however, comparative outcome studies are scarce. 8 9 10 11 12 Various surgical techniques have been reported; the techniques involving neuroma excision and implantation/burying have historically been the most commonly used. However, the outcome of the reported studies is limited by the small sample sizes and nonrandomized designs; therefore, there is still no definitive answer on the effectiveness of each surgical procedure. The purpose of this study is to identify and assess the available information on the outcome of surgical treatment of painful neuromas. Our goals are to determine the overall effectiveness of surgery and find out whether certain surgical procedures are more effective than others.

## Patients and Methods

This retrospective review was initiated after obtaining approval from the institutional review board. Patients at our institute diagnosed with a peripheral neuroma between 1998 and 2018 were identified from their medical records and were reviewed. Inclusion criteria included patients who were operated upon for their painful neuroma in the hand or forearm. Exclusion criteria included patients with neuromas occurring at or proximal to the elbow, those who were managed with nonsurgical methods, and those followed for less than 1 year.

Patient demographics, injury mechanism, hand dominance, associated symptoms, and repair type data were collected. Neuromas were classified according to where they occurred within the hand and forearm, as has been previously described in the literature. 10 Zone I neuromas include those involving the digital nerves and the terminal branches of nerves innervating the dorsum of the hand. Zone II neuromas include those neuromas occurring in the palm of the hand and in the distribution of the dorsal branch of the ulnar nerve. Zone III neuromas include those neuromas occurring in the forearm, including the radial border of the wrist.

Clinical assessment included the identification of a pre- and postoperative Tinel sign, pain visual analog score (VAS), two-point discrimination (2PD), and grip strength, which were obtained from the medical records. The power grip may be affected in neuroma patients secondary to painful inhibition caused by mechanical irritation during grasp. The power grip was evaluated pre- and postoperatively on the same involved side to avoid the variability caused by hand dominance. The power grip was measured using Jamar dynamometer and the results recorded in pounds. Comparison between the pre- and postoperative variables was performed using the two-tailed Student's *t* -test (SPSS software statistical computer package version 26). A *p* -value ≤ 0.05 was considered significant.

## Results

Forty-six patients who met the inclusion criteria were identified. Sixteen patients were lost to follow-up, leaving 29 patients who were treated for 29 individual neuromas. The study population consisted of 11 females (38%) and 18 males (62%) with a mean age of 36 years (range, 17–66 years). Mean follow-up period was 19.07 ± 8.9 months (range, 11–40 months). Mechanisms of injury included clean lacerations (11), crush injuries with finger amputation (11), tumor excision (3), and other trauma or surgery (4). Sixteen neuromas (55%) occurred in the right upper limb. Mean time from initial presentation to the patient's first surgery was 9 months (range, 1–36 months). Preoperative pain evaluations revealed an average rating of severe pain (VAS = 7.4); 3 patients (10%) reported mild pain (VAS = 1–3), 4 patients (14%) reported moderate pain (VAS = 4–6), 20 patients (69%) reported severe pain (VAS = 7–9), and 2 patients (7%) reported intolerable pain (VAS = 10).

All the cases included in this study were symptomatic sensory neuromas. All neuromas, except for one, were end neuromas. Sixteen neuromas occurred in zone I, nine in zone II, and four in zone III. All 16 neuromas in zone I involved the digital nerves. Operative procedures were performed by one surgeon, the senior author (T. M. T.). The surgical policy was dependent on the presence or absence of a distal nerve stump. If a distal nerve stump was present, either primary neurorrhaphy or reconstruction using autogenous nerve grafts, vein conduits, and allografts was performed depending on the defect size. If the distal stump is not retrievable, simple excision, excision and coverage with rotational flap, or excision and transposition of the remaining nerve stump into muscle or bone was performed depending on location of the neuroma and scar condition. Surgical procedures included excision of the neuromas in 10 patients and excision and nerve repair in 19 patients. Of the 10 excised neuromas, simple excision only was performed in 2 patients, excision and coverage with rotational flap in 1 patient, and excision and transposition of the remaining nerve stump into muscle or bone in 6 patients. Of the 19 nerves repaired, 3 underwent primary neurorrhaphy and 4 were reconstructed with autogenous nerve grafts, 9 with vein conduits, and 3 with Axogen graft weave (Axogen Inc., Alachua, Florida, United States).

None of our patients required an additional surgical procedure for adequate treatment of their neuroma. Postoperative pain score revealed that 27 patients (93%) reported mild or no pain, and 2 patients (7%) reported moderate pain. The improvement in postoperative pain score was statistically significant ( *p* ≤ 0.05). Nine patients complained of residual scar hypersensitivity and six patients had residual positive Tinel. 2PD improved from an average of 9.6 ± 4.0 to 6.8 ± 1.0. The improvement in the 2PD was statistically significant ( *p* < 0.05). The 2PD significantly improved only in the excision and reconstruction group from an average of 10.9 to 6.6 mm. The 2PD in the excision-only group did not improve as the difference between the preoperative and postoperative values (1 mm) is negligible and can be attributed to measurement variability ( Table 1 ). The type of procedure performed, excision versus excision and reconstruction, appears to affect functional outcome, in terms of 2PD and grip strength, with nerve repair being superior although the difference was not statistically significant ( Table 1 ). Both types of procedures equally improved postoperative pain by 5 degrees on the VAS. On the other hand, the mechanism of injury, zone of involvement, time from clinical presentation to surgical intervention, or length of follow-up did not have any impact on functional or sensory outcomes ( Tables 2 and 3 ).

**Table 1:** Results according to the surgical procedure

	Neuroma excision and nerve reconstruction		Neuroma excision	
	Pre-op	Post-op	Pre-op	Post-op
Time from injury to onset of symptoms (mo)	7.4		14.5	
VAS	7.1 ± 2.2	1.9 ± 1.9	7.3 ± 2.5	1.5 ± 0.93
2PD (mm)	10.9 ± 4.3	6.6 ± 0.9	8.2 ± 2.7	7.1 ± 1.1
Power grip right (lb)	42.3 ± 21.9	70 ± 27.4	64.8 ± 50.3	73.3 ± 32.2
Power grip left (lb)	36.7 ± 18.9	65 ± 11.8	73.3 ± 32.2	60 ± 15

Abbreviations: 2PD, two-point discrimination; VAS, visual analog scale.

**Table 2:** Results according to the site of neuroma

	Zone I		Zone II		Zone III	
	Pre-op	Pos-top	Pre-op	Post-op	Pre-op	Post-op
Time from injury to onset of symptoms (mo)	10.5 ± 6.9		7.0 ± 9.4		9.8 ± 11	
VAS	7.4 ± 2.2	1.5 ± 1.8	7.3 ± 0.9	2.4 ± 1.8	5.8 ± 3.6	1.6 ± 0.6
2PD (mm)	9.4 ± 2.3	7.0 ± 1.0	11.8 ± 5.6	6.6 ± 1.0	7.7 ± 2.9	6.0
Power grip right (lb)	67.3 ± 28.3	72.0 ± 24.0	48.3 ± 22.3	56.7 ± 20.2	21.7 ± 7.6	110
Power grip left (lb)	44.7 ± 7.8	62.5 ± 6.7	36	65 ± 8.7	–	–

Abbreviations: 2PD, two-point discrimination; VAS, visual analog scale.

**Table 3:** Results according to onset of symptoms

	Time from injury to onset of symptoms (mo)					
	1–5		6–12		>12	
	Pre-op	Post-op	Pre-op	Post-op	Pre-op	Post-op
VAS	7.5 ± 2.4	1.9 ± 1.9	8.2 ± 1.5	0.8 ± 0.9	6.4 ± 2.4	2.1 ± 1.6
2PD (mm)	10.6 ± 4.2	6.5 ± 0.9	11.8 ± 5.1	7.2 ± 1.1	8.6 ± 3.3	6.9 ± 1.0
Power grip right (lb)	46.7 ± 24.4	65.0 ± 24.5	29.0 ± 1.4	–	64.8 ± 50.0	76.0 ± 31.3
Power grip left (lb)	36.0 ± 31.1	55	40.0 ± 28.3	70.0 ± 14.1	43.3 ± 16	62.5 ± 12.9

Abbreviations: 2PD, two-point discrimination; VAS, visual analog scale.

## Discussion

Analysis of our results revealed two findings. The first is that surgical management of painful neuromas led to clinically meaningful improvement of pain, with 93% of patients reporting no or mild postoperative pain regardless of surgical technique employed. This is supported by a recent comparative meta-analysis, which concluded that 77% of patients with neuroma had meaningful improvement of pain regardless of the surgical method used. 13 The second is that excision of a neuroma and nerve reconstruction was marginally better, in terms of 2PD and grip strength, than excision only, excision and transposition of the distal nerve end into muscle or bone, or excision and flap coverage. This is in line with the findings of Guse and Moran, who found that nerve repair had the highest rate of success. 14 Also, Wolvetang et al found lower rates of secondary neuroma surgery in patients undergoing neurorrhaphy with or without nerve graft compared with those undergoing implantation techniques. 15 Although no specific technique proved to be clearly superior, our data demonstrated that surgical intervention should be considered in the treatment algorithm for patients suffering from painful neuroma refractory to conservative management.

The timing of presentation of symptomatic neuroma can vary. It has been proposed that painful symptoms tend to develop within 12 weeks of nerve injury or neuroma treatment. 16

In a study designed to characterize morphologic stages during neuroma development following amputation, Oliveira et al speculated that neuroma treatment and/or prevention strategies might be more successful if targeted at the initial stages of development and not after 28 days following amputation. 17 The average duration between injury and presentation in the present study was 23 ± 8.9 months. The timing of surgery varied due to various nonsurgical methods that were attempted depending on the pain levels and disability.

The location of the neuroma has been suggested as a factor affecting the selection of the treatment method, 10 and several authors have suggested using the zones of the hand to help guide surgical relocation procedures for the neuromas. The contribution of location to the outcome of treatment is also debatable, as some stated pain in zone III is most difficult to treat, 18 whereas others noted worse results in zone I. 19 Analysis in this study revealed that neither the zone nor the involvement of digits had any impact on pain relief or functional recovery.

Previous studies have determined the magnitude of the injury bears no relationship to the development of pain. 12 This is consistent with the current study where the mechanism of injury, sharp laceration or crush, failed to influence postoperative function. It is also worth noting that previous studies confirmed that 20 to 30% of neuromas remain symptomatic regardless of the therapeutic treatment initially applied. 20 In the current study, persistent Tinel sign was present in six patients (20%). However, the presence of postoperative Tinel sign did not have an influence on pain or function and did not necessitate reoperation. It has been suggested that a clearer insight into the benefits from the intervention would be possible only after 36 months of follow-up. 21 Unfortunately, none of our patients had that long of a follow-up.

The principal drawbacks of this study were the retrospective nature and lack of long-term follow-up information. Although patient numbers in this study are large in comparison to those of other studies, larger patient numbers will allow for a multivariate analysis. This will be possible with a prospective multicenter trial.

In conclusion, surgical treatment of hand and forearm neuromas results in significant pain relief and sensory recovery. Nerve repair produces marginally better outcomes compared with neuroma excision only. The mechanism of injury, zone of involvement, time from clinical presentation to surgical intervention, or length of follow-up did not have any impact on functional or sensory outcomes. Further prospective studies are warranted.

## References

[1] Herndon J H Eaton R G Littler J W Management of painful neuromas in the hand J Bone Joint Surg Am 1976 58 03 369 373 1262369

[2] Sunderland S Nerves and Nerve Injuries 2nd ed. Edinburgh Churchill Livingstone 1978

[3] Cravioto H Battista A Clinical and ultrastructural study of painful neuroma Neurosurgery 1981 8 02 181 190 7207783 10.1227/00006123-198102000-00007

[4] Watson J Gonzalez M Romero A Kerns J Neuromas of the hand and upper extremity J Hand Surg Am 2010 35 03 499 510 20193866 10.1016/j.jhsa.2009.12.019

[5] Aasvang E Kehlet H Chronic postoperative pain: the case of inguinal herniorrhaphy Br J Anaesth 2005 95 01 69 76 15531621 10.1093/bja/aei019

[6] Fisher G T Boswick J A Jr Neuroma formation following digital amputations J Trauma 1983 23 02 136 142 6827633 10.1097/00005373-198302000-00012

[7] Gotoda Y Kambara N Sakai T Kishi Y Kodama K Koyama T The morbidity, time course and predictive factors for persistent post-thoracotomy pain Eur J Pain 2001 5 01 89 96 11394926 10.1053/eujp.2001.0225

[8] Nath R Mackinnon S Management of neuromas in the hand Hand Clin 1996 12 04 745 756 8953293

[9] Hazari A Elliot D Treatment of end-neuromas, neuromas-in-continuity and scarred nerves of the digits by proximal relocation J Hand Surg [Br] 2004 29 04 338 350 10.1016/j.jhsb.2004.01.005 15234497

[10] Sood M K Elliot D Treatment of painful neuromas of the hand and wrist by relocation into the pronator quadratus muscle J Hand Surg [Br] 1998 23 02 214 219 10.1016/s0266-7681(98)80177-0 9607662

[11] Atherton D D Fabre J Anand P Elliot D Relocation of painful neuromas in Zone III of the hand and forearm J Hand Surg Eur Vol 2008 33 02 155 162 18443055 10.1177/1753193408087107

[12] Atherton D D Leong J C Anand P Elliot D Relocation of painful end neuromas and scarred nerves from the zone II territory of the hand J Hand Surg Eur Vol 2007 32 01 38 44 17126969 10.1016/j.jhsb.2006.10.013

[13] Poppler L H Parikh R P Bichanich M J Surgical interventions for the treatment of painful neuroma: a comparative meta-analysis Pain 2018 159 02 214 223 29189515 10.1097/j.pain.0000000000001101 PMC5997533

[14] Guse D M Moran S L Outcomes of the surgical treatment of peripheral neuromas of the hand and forearm: a 25-year comparative outcome study Ann Plast Surg 2013 71 06 654 658 22868319 10.1097/SAP.0b013e3182583cf9

[15] Wolvetang N HA Lans J Verhiel S HWL Notermans B JW Chen N C Eberlin K R Surgery for symptomatic neuroma: anatomic distribution and predictors of secondary surgery Plast Reconstr Surg 2019 143 06 1762 1771 30907815 10.1097/PRS.0000000000005664

[16] Koch H Haas F Hubmer M Rappl T Scharnagl E Treatment of painful neuroma by resection and nerve stump transplantation into a vein Ann Plast Surg 2003 51 01 45 50 12838124 10.1097/01.SAP.0000054187.72439.57

[17] Oliveira K MC Pindur L Han Z Bhavsar M B Barker J H Leppik L Time course of traumatic neuroma development PLoS One 2018 13 07 e0200548 30011306 10.1371/journal.pone.0200548 PMC6047790

[18] Foucher G Sammut D Greant P Braun F M Ehrler S Buch N Indications and results of skin flaps in painful digital neuroma J Hand Surg [Br] 1991 16 01 25 29 10.1016/0266-7681(91)90121-4 2007808

[19] Dellon A L Mackinnon S E Treatment of the painful neuroma by neuroma resection and muscle implantation Plast Reconstr Surg 1986 77 03 427 438 2937074 10.1097/00006534-198603000-00016

[20] Nelson A W The painful neuroma: the regenerating axon verus the epineural sheath J Surg Res 1977 23 03 215 221 886855 10.1016/0022-4804(77)90024-5

[21] Colgrove R C Huang E Y Barth A H Greene M A Interdigital neuroma: intermuscular neuroma transposition compared with resection Foot Ankle Int 2000 21 03 206 211 10739150 10.1177/107110070002100304

